# Retroperitoneoscopic nephrectomy for huge autosomal-dominant polycystic kidney disease using morcellator

**DOI:** 10.1590/S1677-5538.IBJU.2017.0258

**Published:** 2018

**Authors:** Dong Sup Lee, Hee Youn Kim, Seung-Ju Lee

**Affiliations:** 1 Department of Urology, The Catholic University of Korea, St. Vincent's Hospital, South Korea

## Abstract

**Introduction and Objectives:**

Nephrectomy is occasionally required due to severe extra-renal symptom(s) such as dyspepsia in patients with autosomal dominant polycystic kidney disease (ADPKD), wherein a large incision is required for specimen extraction. Considering problems such as hernia, wound dehiscence, incidental bowel injury, and poor wound healing in such cases, we would like to present retroperitoneoscopic nephrectomy and morcellation of the kidney as an ideal minimally invasive technique.

**Materials and Methods:**

A 53-year-old man who was undergoing hemodialysis for 6 years due to ADPKD visited the outpatient clinic with a complaint of severe dyspepsia. Kidney length (long axis) was greater than 28 cm. Nephrectomy was the last option to restore his digestive system which was mechanically compressed by an extremely enlarged polycystic kidney. Retroperitoneoscopic nephrectomy was performed using 3 ports. When it was difficult to continue the dissection due to limited space, large cysts were punctured and aspirated to create additional working space. The specimen was extracted by a morcellator (KARL STORZ GmbH & Co. KG, Tuttlingen, Germany) introduced through a 12mm trocar.

**Results:**

Operating time was 230 minutes, wherein the time for morcellation was 52 minutes. No additional incision was required for specimen extraction. He underwent hemodialysis on post-operative days #1 and #3. He was discharged on post-operative day #4 (total hospital stay was 6 days.). Dyspepsia dramatically improved without post-operative complications.

**Conclusions:**

Retroperitoneoscopic nephrectomy is feasible for treatment of ADPKD. By using a morcellator, an additional incision is not required and wound complication would not occur.

## ARTICLE INFO

**Available at: http://www.intbrazjurol.com.br/video-section/20170258_Lee_et_al**

**Int Braz J Urol. 2018; 44 (Video #11): 651-2**

